# Methylation of the epigenetic JMJD2D protein by SET7/9 promotes prostate tumorigenesis

**DOI:** 10.3389/fonc.2023.1295613

**Published:** 2023-11-17

**Authors:** Ruicai Gu, Tae-Dong Kim, Hanlin Jiang, Sook Shin, Sangphil Oh, Ralf Janknecht

**Affiliations:** ^1^ Department of Cell Biology, University of Oklahoma Health Sciences Center, Oklahoma City, OK, United States; ^2^ Department of Pathology, University of Oklahoma Health Sciences Center, Oklahoma City, OK, United States; ^3^ Stephenson Cancer Center, Oklahoma City, OK, United States

**Keywords:** CBLC, gene expression, histone demethylase, KDM4D, PLAGL1, posttranslational modification, prostate cancer

## Abstract

How the function of the JMJD2D epigenetic regulator is regulated or whether it plays a role in prostate cancer has remained elusive. We found that JMJD2D was overexpressed in prostate tumors, stimulated prostate cancer cell growth and became methylated by SET7/9 on K427. Mutation of this lysine residue in JMJD2D reduced the ability of DU145 prostate cancer cells to grow, invade and form tumors and elicited extensive transcriptomic changes. This included downregulation of CBLC, a ubiquitin ligase gene with hitherto unknown functions in prostate cancer, and upregulation of PLAGL1, a transcription factor with reported tumor suppressive characteristics in the prostate. Bioinformatic analyses indicated that *CBLC* expression was elevated in prostate tumors. Further, downregulation of CBLC largely phenocopied the effects of the K427 mutation on DU145 cells. In sum, these data have unveiled a novel mode of regulation of JMJD2D through lysine methylation, illustrated how this can affect oncogenic properties by influencing expression of the *CBLC* gene, and established a pro-tumorigenic role for CBLC in the prostate. A corollary is that JMJD2D and CBLC inhibitors could have therapeutic benefits in the treatment of prostate and possibly other cancers.

## Introduction

Tri- and dimethylation of histone H3 on lysine K9 (H3K9me_3/2_) are prominent epigenetic marks associated with transcriptional repression (1), and changes in these posttranslational modifications are commonly involved in the development of cancer (2). The degree of H3K9 methylation is determined by the opposing actions of histone methyltransferases and demethylases, whose expression or activity is often altered in tumors (3, 4). Jumonji C domain-containing 2D (JMJD2D), also known as lysine demethylase 4D (KDM4D), is one of the enzymes capable of removing methyl moieties from H3K9 (5, 6). Specifically, JMJD2D efficiently demethylates H3K9me_3_ and H3K9me_2_, while its ability to demethylate H3K9me_1_ is at best marginal (7–9). Likewise, JMJD2D demethylates H1.4K26me_3/2_, other marks of repressive chromatin, and again the respective monomethylated state at H1.4K26 is a very poor substrate for JMJD2D (9, 10).

A pro-tumorigenic role of JMJD2D has been most firmly established in colorectal cancer. In particular, JMJD2D overexpression was found in human colorectal tumor specimens; downregulation of JMJD2D compromised colorectal cancer cell proliferation, viability, migration, invasion and xenograft tumor growth; and knockout of *Jmjd2d* compromised chemically induced colitis-associated colon tumor formation as well as spontaneous tumorigenesis in *Apc^min/+^* mice (11–14). Likewise, considerable data suggested that JMJD2D is able to promote the development of gastrointestinal stromal and liver tumors (15–17).

In contrast, although JMJD2D can associate with and thereby modulate DNA-binding transcription factors involved in prostate cancer, namely the androgen receptor and the ETS proteins ETV1 and ETV2 (18–20), no evidence has been presented that JMJD2D could stimulate prostate cancer cells or affect prostate tumor formation. Hence, the role of JMJD2D in prostate tumorigenesis has remained unresolved. Further, if and how JMJD2D is regulated through posttranslational modification is also largely unknown. To narrow these gaps in knowledge, we examined in this report lysine methylation within the JMJD2D protein and in what way this posttranslational modification would affect prostate cancer cells.

## Materials and methods

### Cell lines and human tissue microarray

DU145 (HTB-81), LNCaP (CRL-1740) and 293T (CRL-3216) cells were obtained in authenticated form (American Type Culture Collection, Manassas, VA) and cultured in DMEM plus 10% fetal bovine serum in a humidified atmosphere containing 5% CO_2_ (21, 22). R427 DU145 cells were generated through homology-directed repair using the Alt-R CRISPR-Cas9 system and HDR donor oligos following methods recommended by the manufacturer (Integrated DNA Technologies, Coralville, IA). An AccuMax A302(IV) prostate cancer tissue microarray (Isu Abxis, Seongnam, Korea) encompassing matching normal and cancerous specimens was stained following standard procedures (23, 24) utilizing JMJD2D antibodies (ab93694; Abcam, Waltham, MA) at a dilution of 1:100.

### Immunoprecipitation

Human 293T embryonic kidney cells were seeded onto poly-*L*-lysine coated dishes (25) and a day later transiently transfected by the calcium phosphate coprecipitation method (26, 27). Lysis of transfected cells and immunoprecipitation were then conducted as described before (28, 29). Immunoprecipitates were loaded onto SDS polyacrylamide gels and proteins blotted after separation onto polyvinylidene difluoride membranes (30). Proteins were detected with indicated primary antibodies and respective horseradish peroxidase-coupled secondary antibodies (31) employing enhanced chemiluminescence and exposure to film (32). The following primary antibodies were used: rabbit polyclonal p53-K372me_1_ (ab16033; Abcam, Waltham, MA) and mouse monoclonal Flag M2 (F1804; Sigma-Aldrich, St. Louis, MO).

### *In vitro* methylation assay

Hexahistidine-tagged SET8 and fusion proteins between glutathione *S*-transferase (GST) and JMJD2D or SET7/9 were expressed in *Escherichia coli* (33) and then purified utilizing Ni^2+^-nitrilotriacetic acid agarose or glutathione agarose, respectively (34). Proteins were incubated in 50 mM Tris-HCl (pH 8.5), 5 mM MgCl_2_, 4 mM DTT at 30°C for 2 h in the presence of 1 µM ^3^H-labeled *S*-adenosyl-*L*-methionine. Thereafter, proteins were separated on SDS polyacrylamide gels (35), blotted onto polyvinylidene difluoride membrane (36) and visualized with Ponceau S staining (37). After drying, membranes were sprayed four times every 10 min with EN^3^HANCE (Perkin Elmer, Waltham, MA), dried again and exposed to film at -80°C (38).

### Mass spectrometry

Flag-JMJD2D was transiently coexpressed with Flag-SET7/9 in 293T cells and purified by anti-Flag immunoprecipitation (39). Further procedures were performed at the Mayo Clinic Proteomics Core Facility (Rochester, MN). Briefly, the immunoprecipitates were resolved on an SDS polyacrylamide gel, which was Coomassie stained, and the band corresponding to Flag-JMJD2D excised (40). After trypsin digestion, peptides were analyzed by MS/MS. Calculation of b, b+2H, y and y+2H ion masses was done through the following website: http://db.systemsbiology.net:8080/proteomicsToolkit/FragIonServlet.html; for methylation on K427 or K428, a mass of 14 was added to amino acid #8 or #9, respectively.

### RNA interference and cell assays

Retroviral expression vectors, which were based on the pQCXIH or pSIREN-RetroQ plasmids (Clontech, Mountain View, CA), were transfected together with packaging plasmids into 293T cells (41) and retrovirus collected 24 h and 48 h after transfection (42). Sequences of the utilized JMJD2D shRNAs have been published before (11); for CBLC, shRNA1 and shRNA2 targeted the sequences 5’-GCTGGCCATCATCTTCAGC-3’ and 5’-GTACTGTGGACACATGTAC-3’, respectively. Human DU145 prostate cancer cells were infected with respective retrovirus thrice every 12 h, grown for another 24 h, split and selected with 200 µg/ml hygromycin B or 1-2 µg/ml puromycin for 2-3 days (43). 2500 cells were then seeded into a well of a 96-well plate and cell growth measured with the PrestoBlue cell viability kit (Thermo Fisher, Waltham, MA) as the difference between the absorbance at 570 nm and 595 nm (44). Likewise, 2500 cells were seeded into a well of a 6-well plate and clonogenic activity determined after 10 days by counting crystal violet-stained colonies (45). For invasion assays, cells were treated with 10 µg/ml mitomycin C for 2 h and then their migration through 8 µm filters coated with Matrigel (BioCoat Growth Factor Reduced Matrigel Invasion Chambers #354483; Corning, Durham, NC) measured as described (46). Protein levels were assessed by Western blotting of the antibiotics-selected cells (47) utilizing rabbit polyclonal antibodies for JMJD2D (ab93694; Abcam, Waltham, MA) or mouse monoclonal antibodies for PLAGL1 (NBP2-37343; Novus Biologicals, Centennial, CO), while rabbit polyclonal actin antibodies (A2066; Sigma-Aldrich, St. Louis, MO) were used to control for comparable protein loading.

### Co-immunoprecipitation assays

These assays were performed with transiently transfected 293T cells essentially as described (48, 49). Mouse monoclonal p53 (DO-1; sc-126; Santa Cruz Biotechnology, CA) or Flag M2 (F1804; Sigma-Aldrich, St. Louis, MO) antibodies were employed for immunoprecipitation, while mouse monoclonal Myc 9E10 (M4439; Sigma-Aldrich, St. Louis, MO) or Flag M2 antibodies were used for Western blotting (50).

### Chromatin immunoprecipitation

Cells were crosslinked with 1% formaldehyde for 15 min, after which cells were lysed and sonicated and further processed as described before (51). For immunoprecipitations, rabbit IgG (sc-2027; Santa Cruz Biotechnology, CA) and rabbit polyclonal JMJD2D (ab93694; Abcam, Waltham, MA) antibodies were utilized. Finally, bound promoter fragments were amplified by nested PCR (52) and the resulting DNA fragments visualized through ethidium bromide staining after agarose electrophoresis (53). The PCR primers used were: CBLCfor-401 (5’-GTAGAGACACGGTTTCACCATGTTGG-3’), CBLCfor-316 (5’-CTGGGATTACAGTTGTGAGTCATCGC-3’), CBLCrev-61 (5’-TGCAGGTACCAGTGTCTCCAAAGGGG-3’), CBLCrev-10 (5’-CTCGCCCAGAGTGAAAGGAGAGG-3’), PLAGL1for-434 (5’-CGTTTCTCATGTGTGATTGGGCTCTG-3’), PLAGL1for-398 (5’-CTGGCGGAGACTTCGGCTAGCAGG-3’), PLAGL1rev-49 (5’-GACGGGCTGAATGACAAATGGCAG-3’) and PLAGL1rev-15 (5’- CAGCCGTGTCTAAATCAAGGCTCG-3’).

### Gene expression analyses

RNA was isolated employing Trizol as described (54). After reverse transcription with random primers, RNA was amplified by PCR (55) and relative gene expression was determined with the ΔΔCt method and normalization to levels of *GAPDH* (52). Sequences of oligonucleotides utilized for PCR are listed in Supplementary Methods. RNA sequencing was performed by Novogene (Sacramento, CA) and data analyzed as described before (56).

### Mouse experiments

5-week-old immunocompromised nude male mice (*Fox1^nu^/Fox1^nu^*; #007850; Jackson Laboratory, Bar Harbor, ME) were acclimated for two weeks and then subcutaneously injected through a 27 gauge needle into the right flank (57). For this, 2x10^6^ cells resuspended in 100 µl PBS plus 100 µl growth-factor reduced Matrigel (#354230; Corning, Durham, NC) were employed. Tumor volume (calculated as 0.5 x width x width x length) was measured weekly and tumors were dissected after euthanasia to determine their weight. These mouse experiments were approved by the University of Oklahoma Health Sciences Center Institutional Animal Care and Use Committee.

## Results

### JMJD2D is a substrate of SET7/9

**Figure 1:**
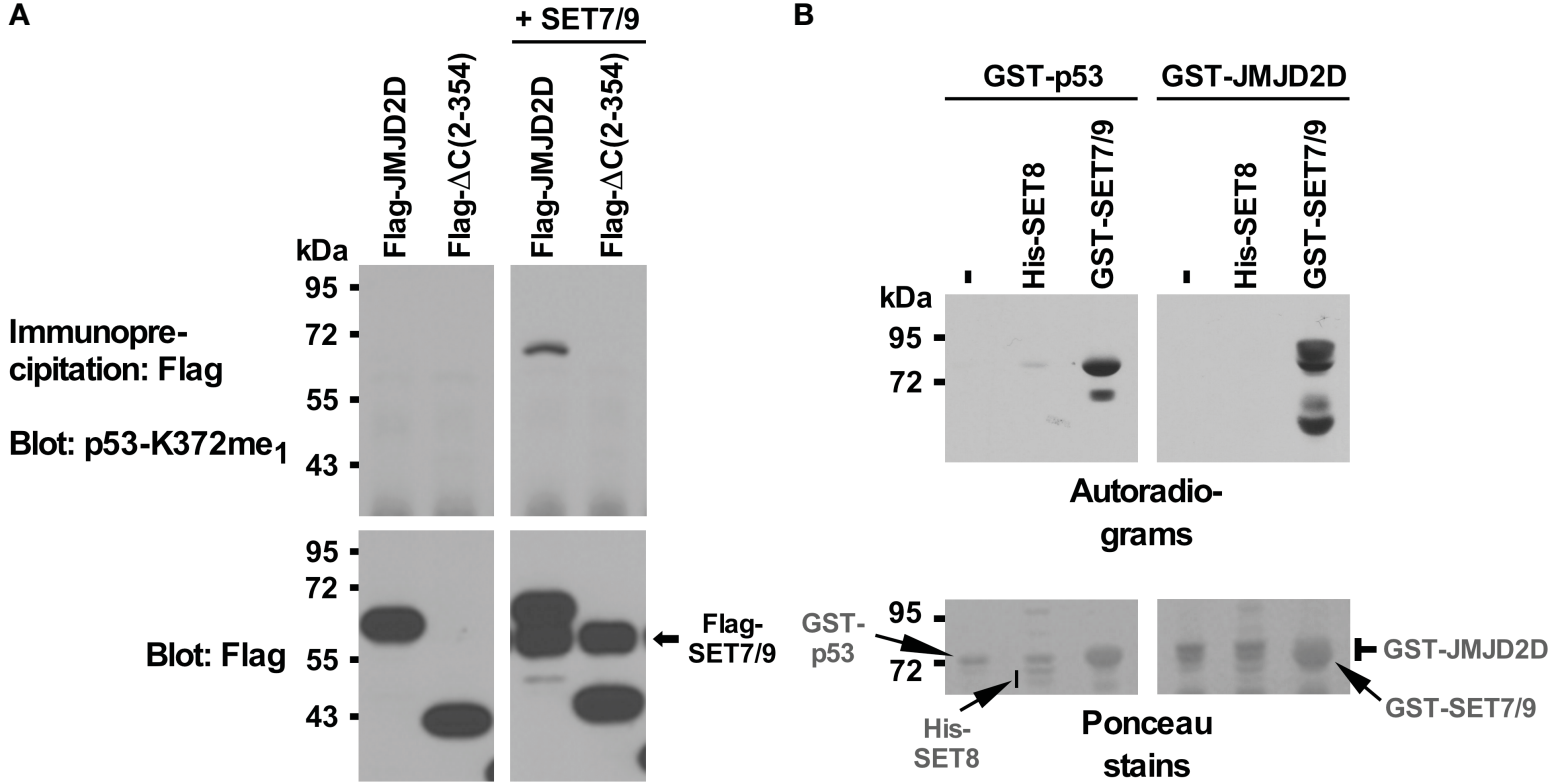
Methylation of JMJD2D by SET7/9. **(A)** Flag-tagged JMJD2D or its C-terminal truncation consisting of amino acids 2-354 was coexpressed with Flag-tagged SET7/9 in 293T cells and immunoprecipitated with anti-Flag antibodies. Methylation was revealed with an antibody that was raised against monomethylated K372 of p53; bottom panels show input levels of the Flag-tagged proteins. **(B)** *In vitro* methylation of purified GST-p53 or GST-JMJD2D by either His-tagged SET8 or GST-tagged SET7/9 utilizing *S*-[methyl-^3^H] adenosyl-*L*-methionine as a methyl donor. Please note that GST-SET7/9 comigrated with GST-p53 and GST-JMJD2D.

Previously, we found that JMJD2D binds to the tumor suppressor p53 (11). It is known that p53 is regulated by methylation, including through SET7/9-mediated methylation of its K372 residue (58). Hence, we wondered if the JMJD2D demethylase would oppose SET7/9-mediated methylation of p53. While we found no evidence for that, we serendipitously uncovered – upon using an antibody recognizing p53 monomethylated on K372 – that JMJD2D itself became methylated when SET7/9 was overexpressed in 293T cells (**Figure 1A**). Furthermore, deleting 169 C-terminal amino acids (see ΔC (2–354) truncation in **Figure 1A**) abrogated this methylation in JMJD2D, suggesting that a lysine residue(s) close to the C-terminus of JMJD2D became methylated by SET7/9.

To demonstrate that SET7/9 directly methylates JMJD2D, we purified respective recombinant proteins and performed *in vitro* methylation assays with radioactive *S*-adenosyl-*L*-methionine as a methyl donor. SET7/9 methylated p53 as expected (**Figure 1B**, left panels) and also JMJD2D (**Figure 1B**, right panels). Moreover, another methyltransferase, SET8, which has been reported to methylate p53 (59), did not utilize JMJD2D as a substrate, whereas a weak activity towards p53 was detectable. These data indicate that SET7/9 can directly methylate JMJD2D.

### Identification of K427 as a methylation site in JMJD2D

**Figure 2:**
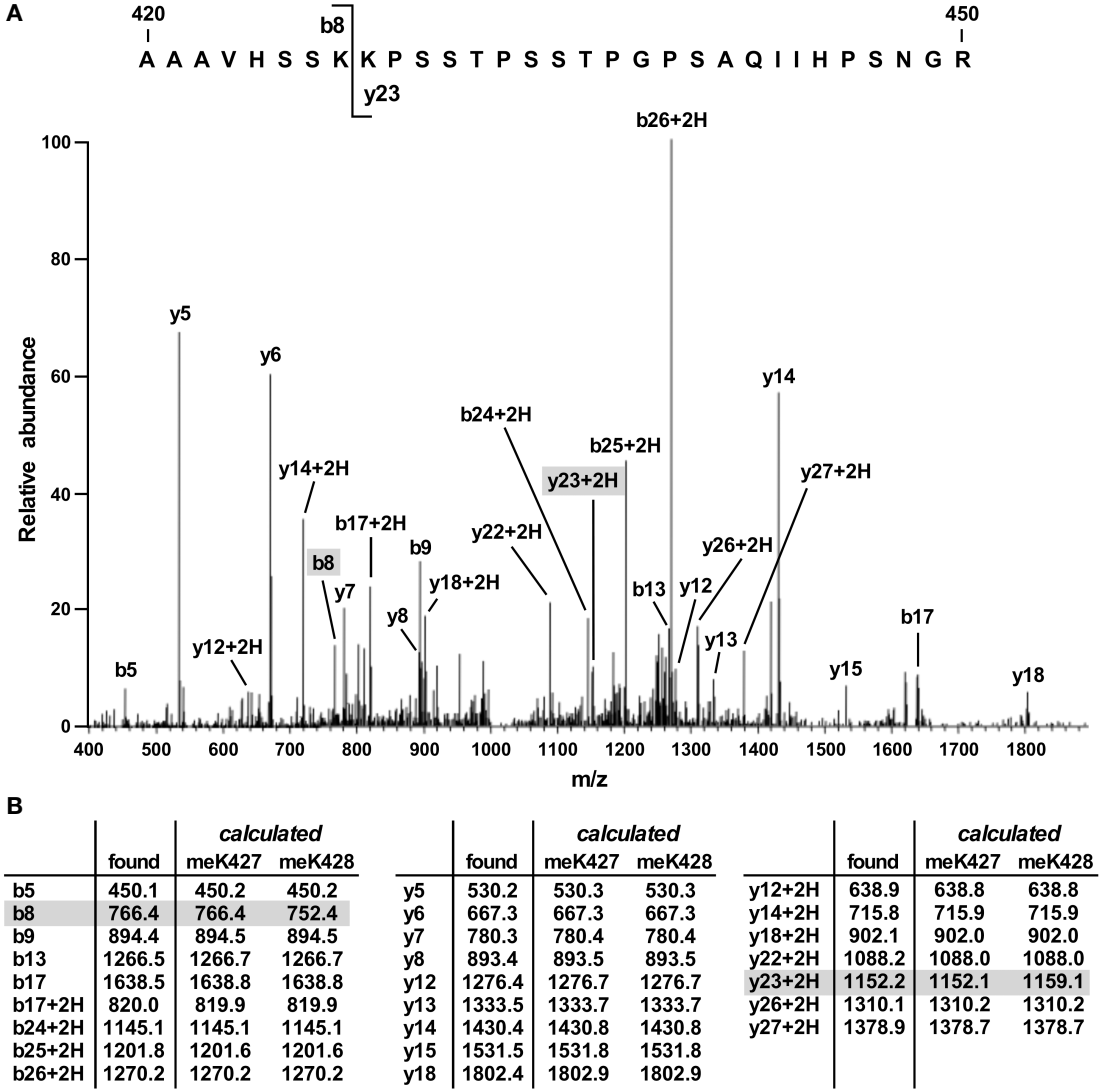
Mass spectrometric identification of methylation on K427. **(A)** Fragmentation pattern of a tryptic peptide (1023.87 [M+3H]^3+^) encompassing amino acids 420-450 of JMJD2D. **(B)** Masses of indicated b, b+2H, y and y+2H ions. Also shown are calculated masses for monomethylation occurring on either K427 or K428; differences were present only for the b8 and y23 + 2H ions.

To identify the site(s) of SET7/9-mediated methylation in JMJD2D, we coexpressed both proteins in 293T cells and then performed mass spectrometry on immunopurified JMJD2D. This revealed only one tryptic peptide consisting of JMJD2D amino acids 420-450 that became monomethylated. This peptide encompassed two lysine residues (K427 and K428). However, the observed fragmentation pattern was only consistent with methylation on K427 (**Figure 2**; see ions b8 and y23 + 2H).

**Figure 3:**
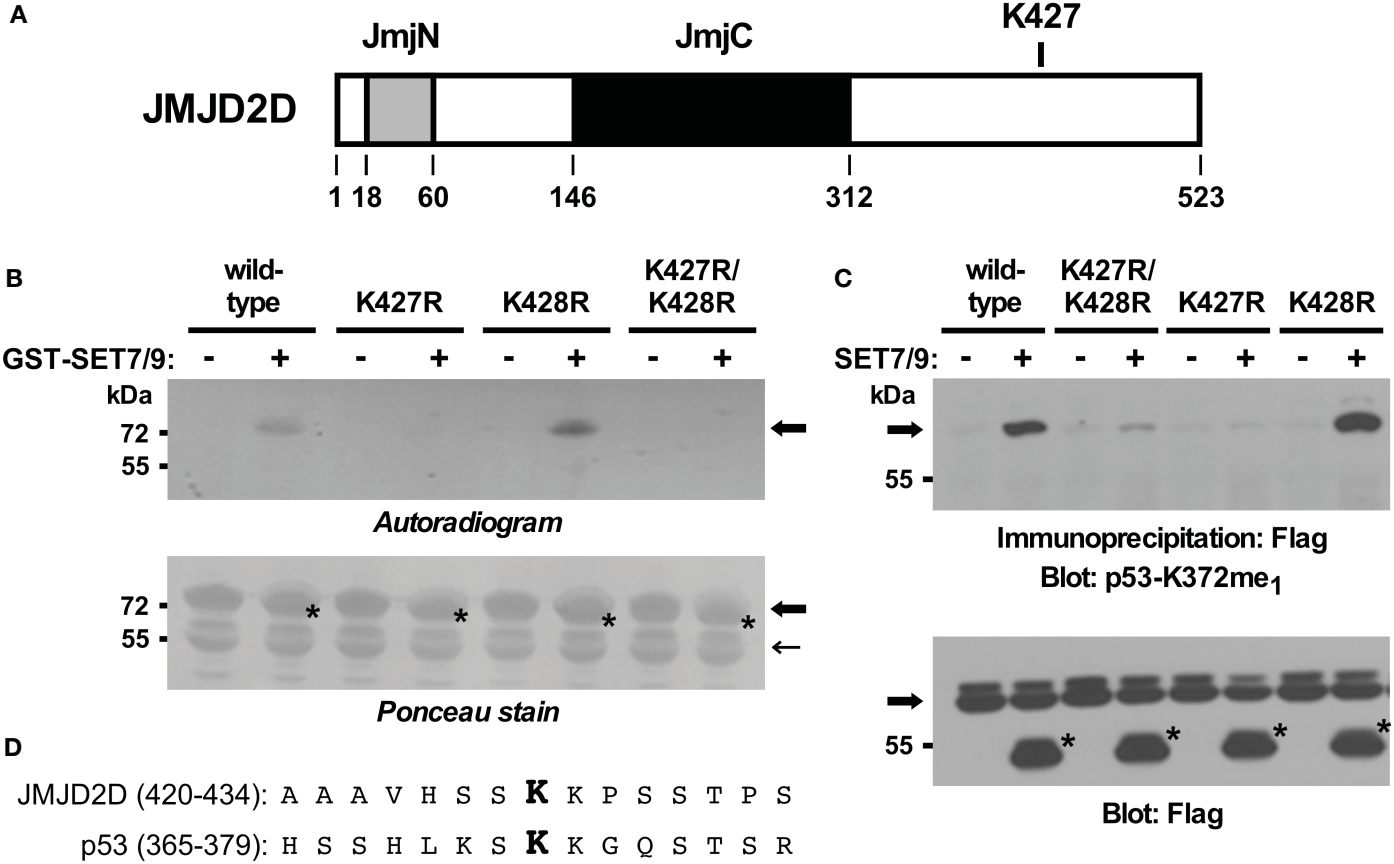
*In vitro* and *in vivo* validation of K427 as a methylation site. **(A)** Scheme of the JMJD2D protein pointing out the catalytic JmjC center and the JmjN domain that also contributes to catalytic activity. **(B)** K427 and/or K428 were mutated to arginine and *in vitro* SET7/9-mediated methylation of respective GST-JMJD2D fusion proteins analyzed. Bold arrows mark GST-JMJD2D fusion proteins, a light arrow a degradation product of GST-JMJD2D (likely a C-terminal truncation no longer containing K427), and asterisks GST-SET7/9. Please note that the GST fusions of JMJD2D and SET7/9 comigrated. **(C)** Wild-type Flag-JMJD2D or indicated mutants thereof were expressed in 293T cells in the absence or presence of Flag-tagged SET7/9. After anti-Flag immunoprecipitation, methylation was examined by blotting with p53-K372me_1_ antibodies. Arrow points at Flag-tagged JMJD2D (wild-type or mutated) and asterisks mark Flag-SET7/9. **(D)** Alignment of JMJD2D amino acids 420-434 with p53 amino acids 365-379. Respective monomethylated lysine residues K427 and K372 are highlighted in bold.

To validate this inference, we also generated mutated GST-JMJD2D fusion proteins and examined their ability to become methylated by SET7/9 *in vitro*. In contrast to wild-type JMJD2D, the K427R mutant was no longer methylated (**Figures 3A, B**). As a control, a K428R mutant was still utilized as a substrate by SET7/9, while a K427R/K428R double mutant was not. Similarly, we assessed *in vivo* methylation with JMJD2D point mutants. Again, we observed that mutation of K427, but not K428, to arginine compromised SET7/9-dependent methylation (**Figure 3C**). Please note that amino acids surrounding JMJD2D-K427 and p53-K372 are similar (**Figure 3D**), potentially explaining why the antibody raised against monomethylated K372 of p53 cross-reacted with JMJD2D when monomethylated on K427. Altogether, our data demonstrate that K427 in JMJD2D can be methylated by SET7/9.

### Methylation of K427 affects the function of JMJD2D in prostate cancer cells

**Figure 4:**
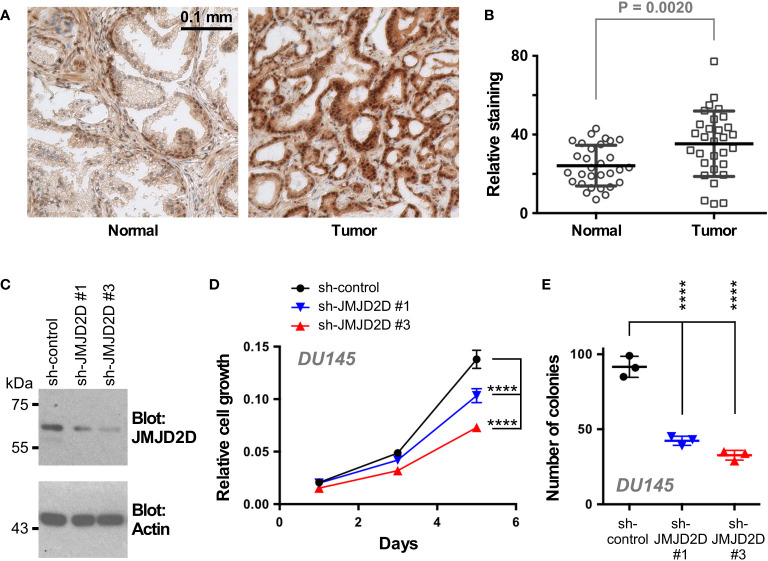
Role of JMJD2D in prostate cancer cells. **(A)** Representative staining for JMJD2D in normal and cancerous prostate tissue. **(B)** Quantitation of JMJD2D staining in 30 matching prostate specimens; paired, two-tailed t test. **(C)** Downregulation of JMJD2D with two different shRNAs was assessed by Western blotting. **(D)** Corresponding cell growth assay. Statistical significance was assessed with two-way ANOVA (Tukey’s multiple comparison test; n=3). **(E)** Clonogenic activity; one-way ANOVA (Tukey’s multiple comparison test; n=3). ****P<0.0001.

To examine a potential role of JMJD2D in prostate cancer, we first stained a human tissue microarray composed of matching normal and cancerous prostate tissue with JMJD2D antibodies in order to answer if JMJD2D is expressed in the human prostate. Indeed, JMJD2D expression was observed in both normal prostates and adenocarcinomas; notably, its expression was significantly enhanced in tumors (**Figures 4A, B**). Further, downregulation of JMJD2D with two different shRNAs resulted into less growth and clonogenic activity of DU145 human prostate cancer cells (**Figures 4C–E**), and similarly JMJD2D downregulation diminished the growth of human LNCaP prostate cancer cells (**Supplementary Figure 1**). These results suggest that JMJD2D is a promoter of prostate tumorigenesis.

**Figure 5:**
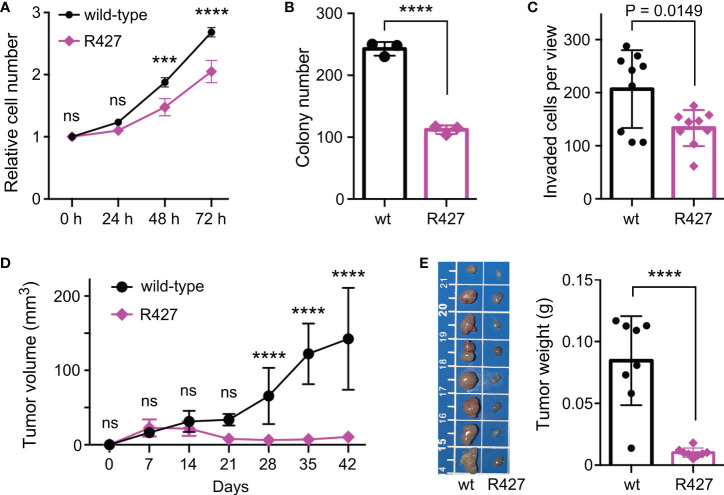
Phenotypic changes in homozygous R427 DU145 knockin cells. **(A)** Cell growth assay. Statistical significance was assessed with two-way ANOVA (Sidak’s multiple comparison test; n=3). **(B)** Clonogenic activity; unpaired, two-tailed t test (n=3). **(C)** Cell invasion; unpaired, two-tailed t test (n=9). **(D)** Tumor growth after subcutaneous injection of indicated cells into nude mice. Statistical significance was assessed with two-way ANOVA (Sidak’s multiple comparison test; n=8). **(E)** Corresponding tumors dissected after 42 days of growth (scale bar in cm) and their weights; unpaired, two-tailed t test (n=8). ***P<0.001; ****P<0.0001; ns, not significant.

To determine how methylation of K427 would affect JMJD2D’s function, we examined if the R427 mutation would tamper the interaction of JMJD2D with two of its prostate cancer-relevant interaction partners, the tumor suppressor p53 and the ETS protein ETV1 (11, 19). However, wild-type and R427 JMJD2D did not noticeably differ in their abilities to form complexes with p53 or ETV1 (**Supplementary Figure 2**). We also performed CRISPR/Cas9-mediated mutagenesis in DU145 prostate cancer cells in order to replace K427 by an arginine residue and were able to obtain respective homozygous R427 DU145 cells (**Supplementary Figures 3A, B**). Notably, this did not lead to a change in H3K9me_3_ levels (**Supplementary Figure 3C**). Then, we compared wild-type to R427 DU145 prostate cancer cells and found that R427 cells displayed significantly reduced growth, clonogenic activity and invasion potential *in vitro* (**Figures 5A–C**). Further, we injected these cells subcutaneously into nude mice and observed that the R427 cells were also compromised in their ability to form tumors *in vivo* (**Figures 5D, E**). Collectively, these data indicate that K427 methylation stimulates JMJD2D’s oncogenic properties in DU145 cells.

### Transcriptome changes in R427 cells

**Figure 6:**
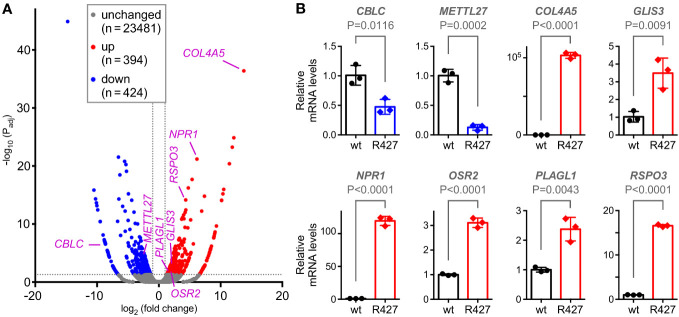
Transcriptomic changes induced by the R427 mutation. **(A)** Volcano plot showing differential gene expression (P_adj_<0.05 and |fold change|>2) in R427 compared to wild-type DU145 prostate cancer cells. **(B)** Validation of the changed expression of the indicated 8 genes by quantitative RT-PCR; unpaired, two-tailed t test (n=3).

To understand how mutation of K427 affects DU145 cell physiology, we performed RNA sequencing. Compared to wild-type DU145 cells, the R427 cells displayed roughly each 400 significantly up- and downregulated genes (**Figure 6A**). From those, we selected 8 genes (*CBLC*, *METTL27*, *COL4A5*, *GLIS3*, *NPR1*, *OSR2*, *PLAGL1*, *RSPO3*) based on their known or their unanticipated function in prostate cancer (see below and Discussion) and corroborated their altered expression by quantitative RT-PCR (**Figure 6B**). Bioinformatic analyses (**Supplementary Figure 4A**) indicated that genes upregulated in R427 cells (*COL4A5*, *GLIS3*, *NPR1*, *OSR2*, *PLAGL1*, *RSPO3*) were all significantly downregulated in human prostate adenocarcinomas, while genes downregulated in R427 cells were either significantly higher expressed (*CBLC*) or trending towards enhanced expression (*METTL27*) in prostate tumors. This would be consistent with the notion that methylation of JMJD2D could promote prostate cancer development through upregulation of *CBLC* and *METTL27* as well as through downregulation of *COL4A5*, *GLIS3*, *NPR1*, *OSR2*, *PLAGL1* and *RSPO3*.

**Figure 7:**
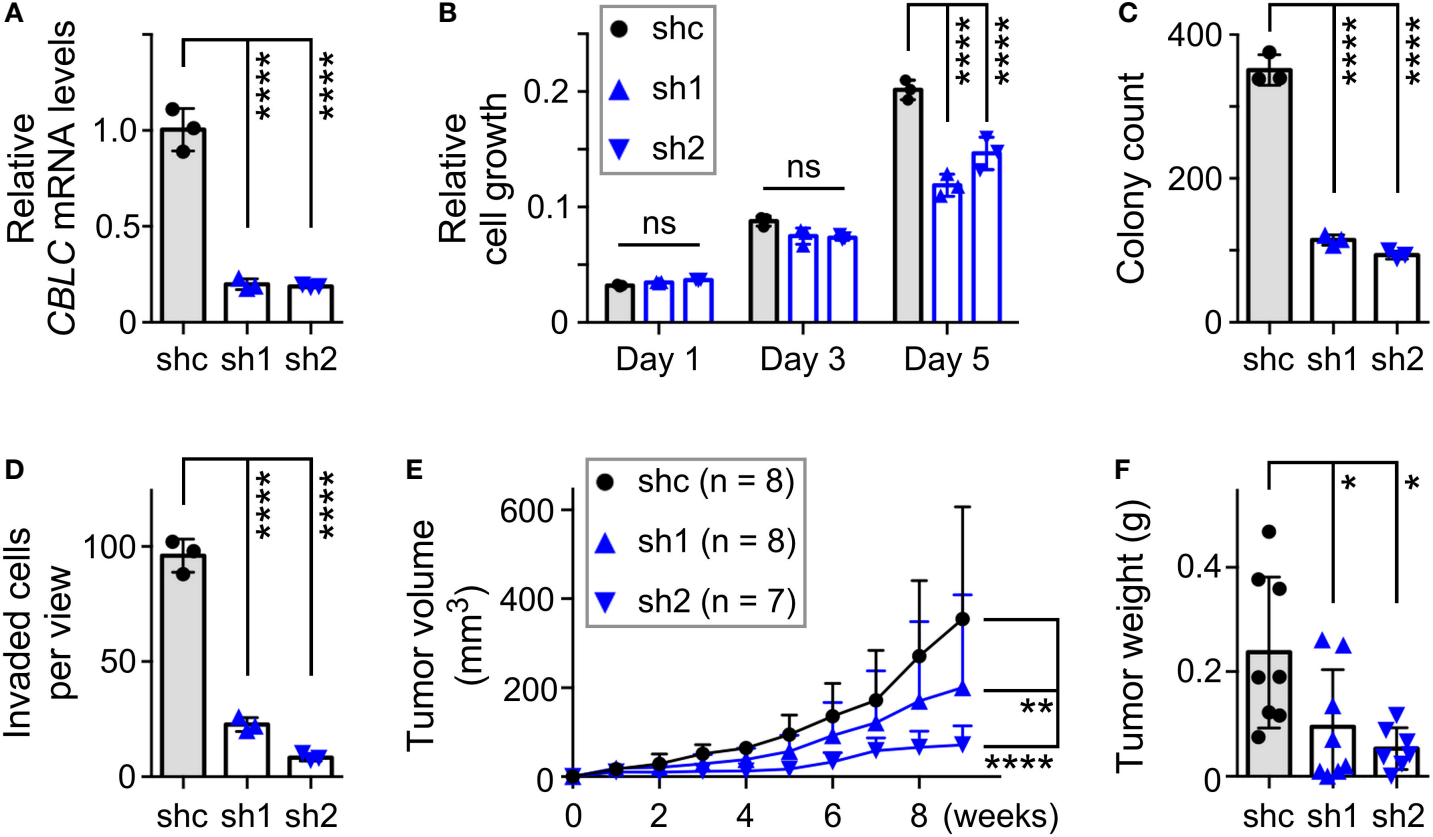
Pro-tumorigenic function of CBLC in DU145 prostate cancer cells. **(A)** RT-PCR showing that expression of two different CBLC shRNAs (sh1, sh2) led to reduction of *CBLC* mRNA levels compared to control shRNA (shc); one-way ANOVA (Tukey’s multiple comparison test; n=3). **(B)** Corresponding cell growth assay. Statistical significance was assessed with two-way ANOVA (Tukey’s multiple comparison test; n=3). **(C)** Respective clonogenic activity and **(D)** cell invasion; one-way ANOVA (Tukey’s multiple comparison test; n=3). **(E)** Subcutaneous tumor growth in nude mice. Statistical significance was assessed with two-way ANOVA (Tukey’s multiple comparison test). **(F)** Corresponding tumors dissected after 9 weeks of growth; one-way ANOVA (Tukey’s multiple comparison test). *P<0.05; **P<0.01; ****P<0.0001; ns, not significant.

Our interest was particularly piqued by *CBLC* (cbl proto-oncogene C) since it is robustly expressed in normal prostate tissue (60, 61), yet its relationship with prostate cancer has remained unknown. Moreover, chromatin immunoprecipitation experiments indicated that JMJD2D interacted with the *CBLC* gene promoter (**Supplementary Figure 4B**), strongly suggesting that JMJD2D can directly regulate *CBLC* transcription. To study the role of *CBLC* in DU145 cells, we downregulated it with two different shRNAs (**Figure 7A**). This resulted into significantly reduced cell growth, clonogenic activity, invasion and tumor formation (**Figures 7B–F**), which is similar to the phenotype of the R427 mutation (see **Figure 5**). Our data have thereby identified *CBLC* as a novel promoter of prostate cancer and to be potentially one of the seminal genes regulated by methylated JMJD2D.

## Discussion

The physiological role of JMJD2D in prostate cancer has hitherto remained elusive. Our data revealed that JMJD2D is overexpressed in prostate tumors and can support the growth of prostate cancer cells. Moreover, methylation of K427 appears to be seminal for JMJD2D’s impact on oncogenic characteristics like *in vitro* cell growth, clonogenic activity and invasion as well as *in vivo* tumor formation. This could explain in part why SET7/9 reportedly stimulates prostate cancer cells, namely through JMJD2D methylation, albeit this may additionally involve the methylation of the androgen receptor (62, 63). Since SET7/9 is expressed in various tumors (64), it is likely that JMJD2D methylation on K427 will also play a role in malignancies other than prostate cancer that JMJD2D is involved in. Interestingly, JMJD2D stimulates the self-renewal of liver cancer stem-like cells (17), implying that methylated JMJD2D may likewise affect stem-like cells in prostate tumors. In addition, JMJD2D has been reported to promote hepatic fibrogenesis or animal cloning through somatic cell nuclear transfer (65, 66), suggesting that K427 methylation of JMJD2D could also affect such other biological processes. However, these suppositions are in need of experimental validation.

JMJD2D is part of the JMJD2 family that is comprised of up to 6 members (5, 6). JMJD2A-C are ~130 kDa proteins, while JMJD2D-F are roughly half the size and only highly homologous within their N-terminal portions to JMJD2A-C (67). Moreover, even within the domains contributing to catalytic activity, JMJD2D is slightly, but distinctively, different in its structure, resulting in the inability of demethylating H3K36me_3/2_ which is in contrast to JMJD2A-C (68, 69). The K427 methylation site of JMJD2D resides within its C-terminal region that is not well conserved among the JMJD2 family members. Accordingly, alignment of the JMJD2 protein sequences shows that there is no lysine homologous to K427 in other JMJD2 proteins except for JMJD2B (**Supplementary Figure 5**). However, this respective lysine at residue 608 of JMJD2B does not appear to become methylated by SET7/9 (our unpublished results). Thus, methylation of JMJD2D on K427 provides an opportunity for SET7/9 to regulate only one of the six JMJD2 proteins in a unique manner.

JMJD2D is a transcriptional cofactor (5, 6) and accordingly, we found that mutation of K427 resulted into an altered gene expression pattern in DU145 cells. In particular, methylation of JMJD2D stimulated *CBLC* gene expression. CBLC is a E3 ubiquitin ligase that was reported to cause the destabilization of tyrosine kinases such as EGFR, SRC and RET (70–72). As these kinases are proto-oncoproteins, CBLC may serve a tumor suppressive function similar to its close homologs, CBL and CBLB (73, 74). However, this is contradicted by a study showing that CBLC promoted lung adenocarcinoma development through activating EGFR. This was likely due to the fact that CBLC imposed EGFR polyubiquitylation through K6 and K11 of ubiquitin, which was in contrast to CBL that did so through K63 and K48. Thereby, CBLC promoted cellular trafficking and recycling of the activated EGFR receptor and also precluded the CBL-mediated destabilization of EGFR (75). In addition, CBLC may not always lead to the destabilization of RET, but cause exactly the opposite in the absence of an interacting protein, CD2AP (72), thereby potentially also stimulating tumor formation. And similar to our discovery that *CBLC* is upregulated in prostate tumors, *CBLC* has been shown to be overexpressed in lung adenocarcinomas (75, 76), furthering the notion that CBLC is capable of promoting tumorigenesis. Consistently, we observed that CBLC downregulation phenocopied the anti-oncogenic effects of the R427 mutation in DU145 cells, establishing for the first time that CBLC exerts tumor promoting activities in prostate cancer cells.

The R427 mutation of JMJD2D led not only to reduced expression of *CBLC*, but also of *METTL27*, which encodes for a methyltransferase (77) with unknown biological function. But in contrast to CBLC, METTL27 downregulation had no impact on DU145 cell growth or clonogenic activity (**Supplementary Figures 6A–C**). On the other hand, we focused on six genes (*COL4A5*, *GLIS3*, *NPR1*, *OSR2*, *PLAGL1* and *RSPO3*) that were significantly downregulated in human prostate tumors as well as upregulated in DU145-R427 cells, suggesting that these genes might negatively interfere with prostate tumorigenesis. However, the literature does not support a tumor suppressing role for the *GLIS3* transcription factor gene, as *GLIS3* reportedly exerted tumor-promoting activities in melanoma and breast cancer (78, 79). Similarly, *RSPO3*, which encodes for a secreted signaling molecule and potentiates Wnt/β-catenin signaling, is characterized as a tumor promoter (80, 81). For the atrial natriuretic peptide receptor gene *NPR1*, a potential role in prostate cancer is controversial: while atrial natriuretic peptide displayed anti-cancer activity in prostate adenocarcinoma cells (82), *NPR1* ablation caused apoptosis that suggests a tumor-promoting role for *NPR1* by facilitating cancer cell survival (83). And in case of *OSR2*, which encodes for a transcription factor that may modulate the transition between the epithelial and mesenchymal state (84), we did not find any effect of its overexpression on DU145 cell growth, albeit a slight reduction of clonogenic activity was noticeable (**Supplementary Figures 6D–F**). All this suggests that it is unlikely that JMJD2D impinges in a meaningful way on the oncogenic potential of prostate cancer cells through modulating the expression of *GLIS3*, *RSPO3*, *NPR1* or *OSR2*.

But published data about *COL4A5*, which encodes for one of the six subunits of type IV collagen that is a major component of the basement membrane, are in accordance with a potential tumor suppressing function: type IV collagen was shown to be reduced in the basement membrane of prostate tumors (85) and loss of COL4A5 was particularly noted in invasive prostate carcinomas (86). Moreover, the transcription factor PLAGL1 is considered to be a tumor suppressor (87) and its downregulation in prostate tumors is possibly due to epigenetic silencing (88, 89). Consistently, PLAGL1 overexpression in DU145 cells resulted into reduced cell growth and clonogenic activity (**Supplementary Figures 6G–I**). Further, JMJD2D appears to interact with the *PLAGL1* gene promoter (**Supplementary Figure 4C**), which is consistent with the notion that JMJD2D is involved in *PLAGL1* transcriptional regulation. Collectively, these data indicate that JMJD2D likely affects prostate tumorigenesis through modulating transcription of not only *CBLC*, but also other genes, including *PLAGL1* and *COL4A5*.

Apart from transcriptional regulation, JMJD2D has other functions such as the modulation of the DNA damage response and DNA replication (90, 91). If and how K427 methylation influences these activities of JMJD2D and whether this would affect prostate tumorigenesis remains to be studied. Lastly, *Jmjd2d* knockout mice were viable and without obvious pathological defects (92). This suggests that targeting JMJD2D in prostate cancer, either through blocking its catalytic center or precluding its methylation on K427, could be a relatively side effect-free way of therapy. Similarly, *Cblc* knockout mice did not display any obvious deficiencies (93), highlighting that blocking CBLC function might also be leveraged for the treatment of prostate cancer.

## Data availability statement

The datasets presented in this study can be found in online repositories. The names of the repository/repositories and accession number(s) can be found below: https://www.ncbi.nlm.nih.gov/, BioProject PRJNA793858.

## Ethics statement

Ethical approval was not required for the studies on humans in accordance with the local legislation and institutional requirements because only commercially available established cell lines were used. The animal study was approved by Institutional Animal Care and Use Committee, OUHSC. The study was conducted in accordance with the local legislation and institutional requirements.

## Author contributions

RG: Conceptualization, Formal analysis, Investigation, Methodology, Visualization, Writing – original draft, Writing – review & editing. T-DK: Conceptualization, Formal analysis, Investigation, Methodology, Writing – review & editing. HJ: Formal analysis, Investigation, Writing – review & editing. SS: Formal analysis, Methodology, Writing – review & editing. SO: Formal analysis, Methodology, Supervision, Writing – review & editing. RJ: Conceptualization, Formal analysis, Funding acquisition, Investigation, Methodology, Project administration, Supervision, Visualization, Writing – original draft, Writing – review & editing.
