# Supplementary material for: Methylation of the epigenetic JMJD2D protein by SET7/9 promotes prostate tumorigenesis
Source: Front Oncol. 2023 Nov 17;13:1295613. doi: 10.3389/fonc.2023.1295613 (PMC10690936; doi:10.3389/fonc.2023.1295613)
Supplement: Supplementary file 1 [file DataSheet_1.pdf]

SUPPLEMENTARY MATERIAL

Methylation of the Epigenetic JMJD2D Protein by SET7/9 Promotes Prostate Tumorigenesis

Ruicai Gu, Tae-Dong Kim, Hanlin Jiang, Sook Shin, Sangphil Oh, Ralf Janknecht

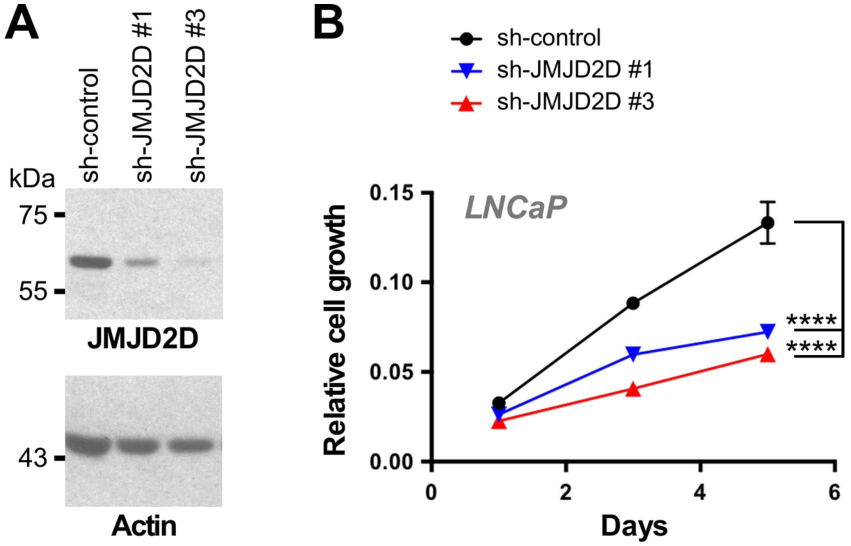

Supplementary Figure 1. Downregulation of JMJD2D compromised LNCaP prostate cancer cell growth. (**A**) Cells were infected twice with retrovirus expressing indicated shRNAs and selected for 3 days with 1 µg/ml puromycin before experimental use. Downregulation of JMJD2D with the two different shRNAs was then assessed by Western blotting. (**B**) Corresponding cell growth assay. Statistical significance was assessed with two-way ANOVA (Tukey's multiple comparison test; n=3). ****, P<0.0001.

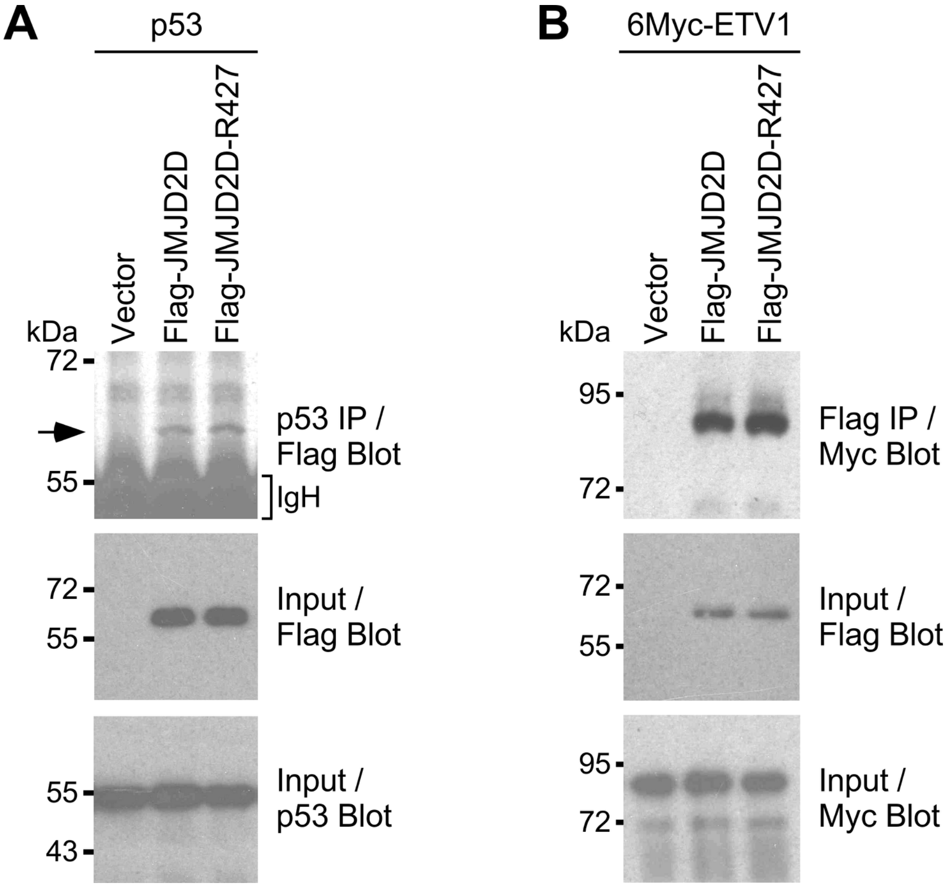

Supplementary Figure 2. Interaction of JMJD2D with p53 and ETV1 in 293T cells determined by co-immunoprecipitation assays. (**A**) Flag-JMJD2D (wild-type or R427 mutant) was coexpressed with p53. After immunoprecipitation with anti-p53 antibodies, coprecipitated JMJD2D was revealed by anti-Flag Western blotting (top panel); arrow points at JMJD2D. IgH, immunoglobulin heavy chain. The bottom two panels show input levels for Flag-tagged JMJD2D or for p53. (**B**) Similarly, co-immunoprecipitation of 6Myc-tagged ETV1 with Flag-tagged JMJD2D.

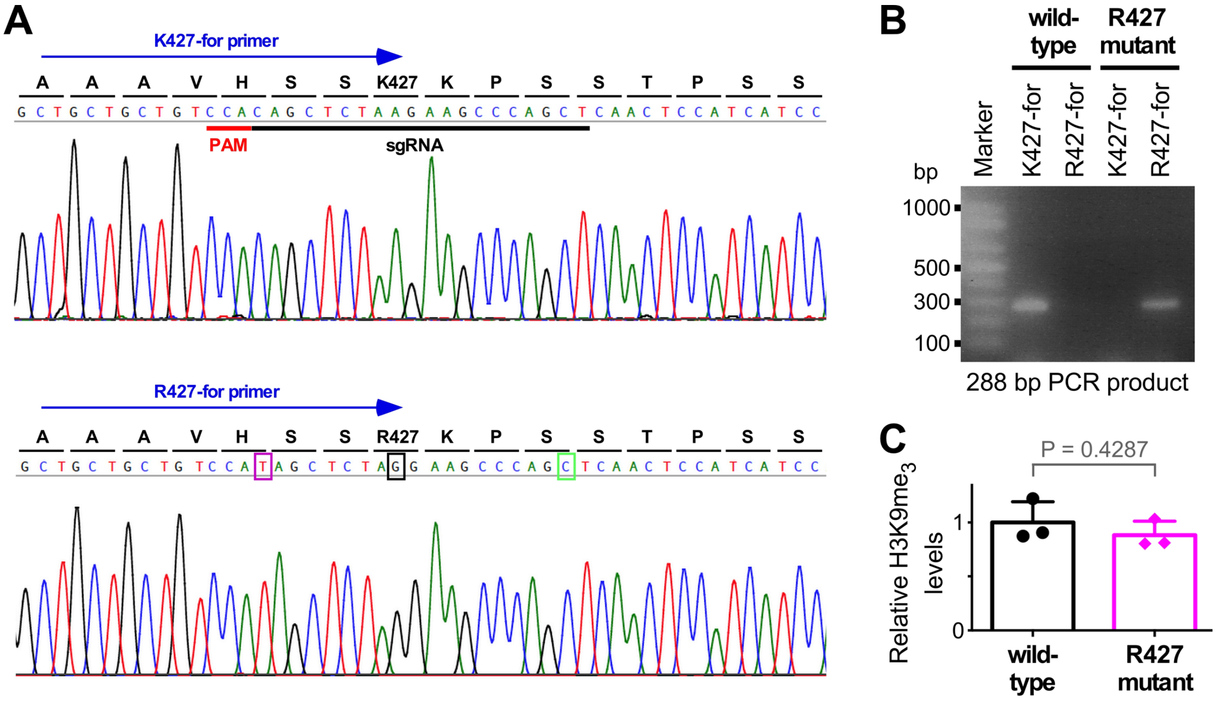

Supplementary Figure 3. CRISPR-modified DU145 prostate cancer cells. (**A**) DNA sequencing of genomic DNA. Shown are wild-type cells (top) and R427 cells (bottom). The sequence of the sgRNA utilized in creating the knockin cells is underlined, and the PAM sequence highlighted in red color. The positions of two silent mutations that were introduced into the donor oligonucleotide to prevent re-cutting by Cas9 are boxed on the bottom (CA<u>C</u> to CA<u>T</u> for H424 in magenta color, and AG<u>C</u> to AG<u>T</u> for S430 in green color); however, only one of these silent mutations (magenta color) was found in the genomic DNA of R427 cells, suggesting that recombination between the Cas9-cut genomic DNA and the donor oligonucleotide occurred upstream of the second silent mutation. (**B**) PCR with the JMJD2D-2350-rev primer and either the K427-for primer or R427-for primer on wild-type and R427 cells. The latter two primers are shown in panel A and were designed to match either the wild-type (K427) or the R427 mutant, thus specifically amplifying only one or the other. See further experimental details in the *Supplementary Methods* section below. (**C**) H3K9me$_3$ levels in wild-type and R427 DU145 cells were determined by quantitative Western blotting and normalized to total histone H3 levels; unpaired, two-tailed t test (n=3).

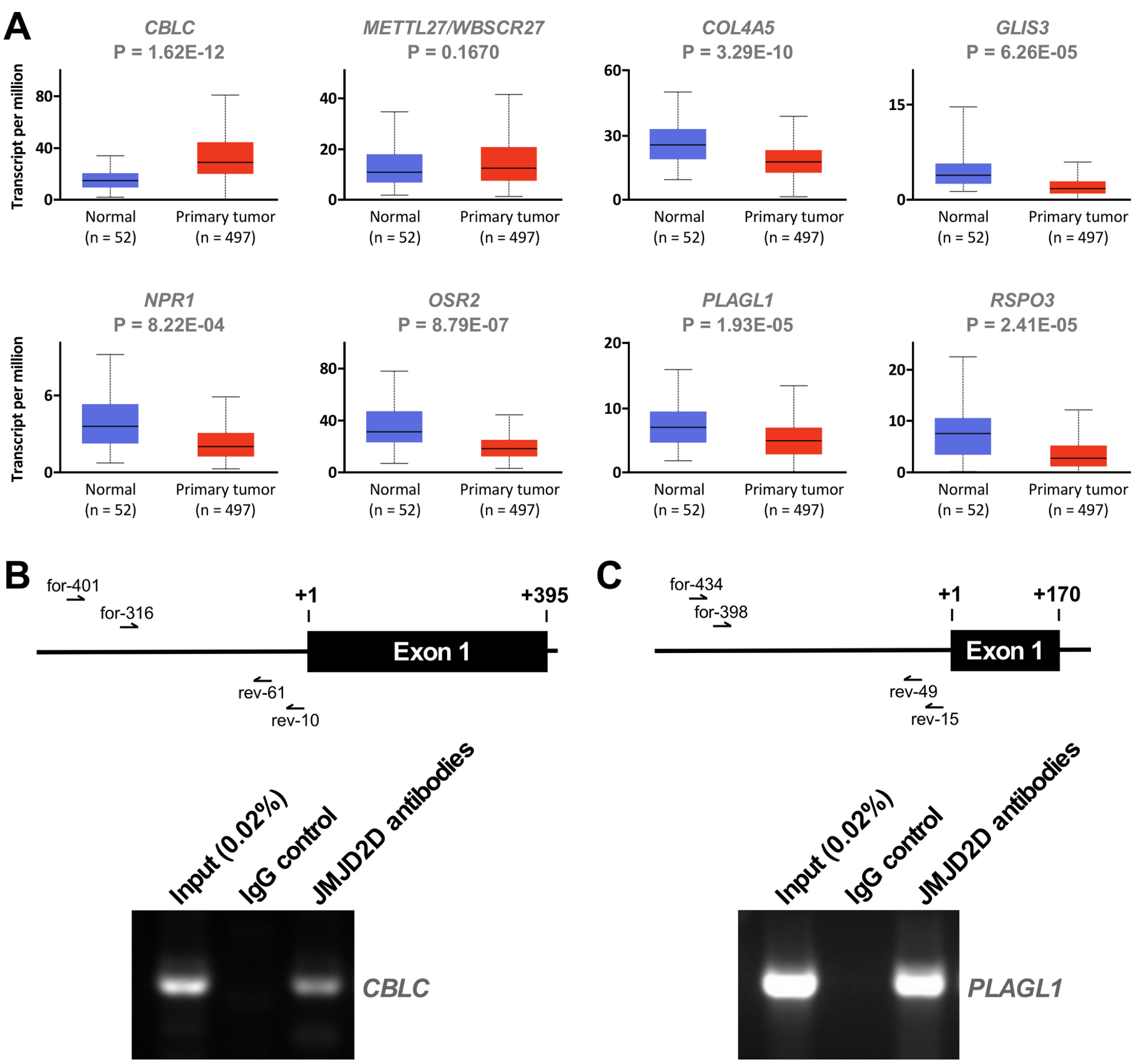

Supplementary Figure 4. (**A**) Gene expression changes (unpaired t test) in prostate adenocarcinomas compared to normal prostate tissue that were found in *The Cancer Genome Atlas* (*TCGA*). Analysis was done through *The University of Alabama at Birmingham Cancer Data Analysis Portal* (UALCAN; https://ualcan.path.uab.edu/). (**B**) Chromatin immunoprecipitation assay for the *CBLC* promoter. In the top sketch, the location of the forward and reverse primers utilized for nested PCR are indicated within the *CBLC* promoter. Transcriptional start site is defined as +1 and is according to the NCBI sequence NM_012116.4. Bottom shows an ethidium bromide-stained agarose gel with the amplified *CBLC* promoter fragment. (**C**) Likewise for *PLAGL1* (NM_006718.5).

						427						
JMJD2D (422-433):	A	V	H	S	S	K	K	P	S	S	T	P
JMJD2E (426-437):	R	V	L	L	P	S	T	G	S	W	G	S
JMJD2F (415-426):	W	V	L	P	P	S	T	G	R	W	G	P
JMJD2A (588-599):	E	E	N	K	K	S	K	G	R	R	Q	P
JMJD2B (603-614):	L	K	M	E	I	K	K	S	R	R	H	P
JMJD2C (563-574):	G	E	N	K	T	S	K	S	W	R	H	P

Supplementary Figure 5. Sequence alignment of human JMJD2 proteins around the methylated K427 residue of JMJD2D. The shown 12 amino acids of all six JMJD2 proteins were derived from NCBI accession numbers NP_060509.2 (JMJD2D), NP_001155102.1 (JMJD2E), NG_012275.2 (from the longest predicted open reading frame consisting of 638 amino acids encoded in this *JMJD2F* pseudogene), NP_055478.2 (JMJD2A), NP_055830.1 (JMJD2B) and NP_055876.2 (JMJD2C).

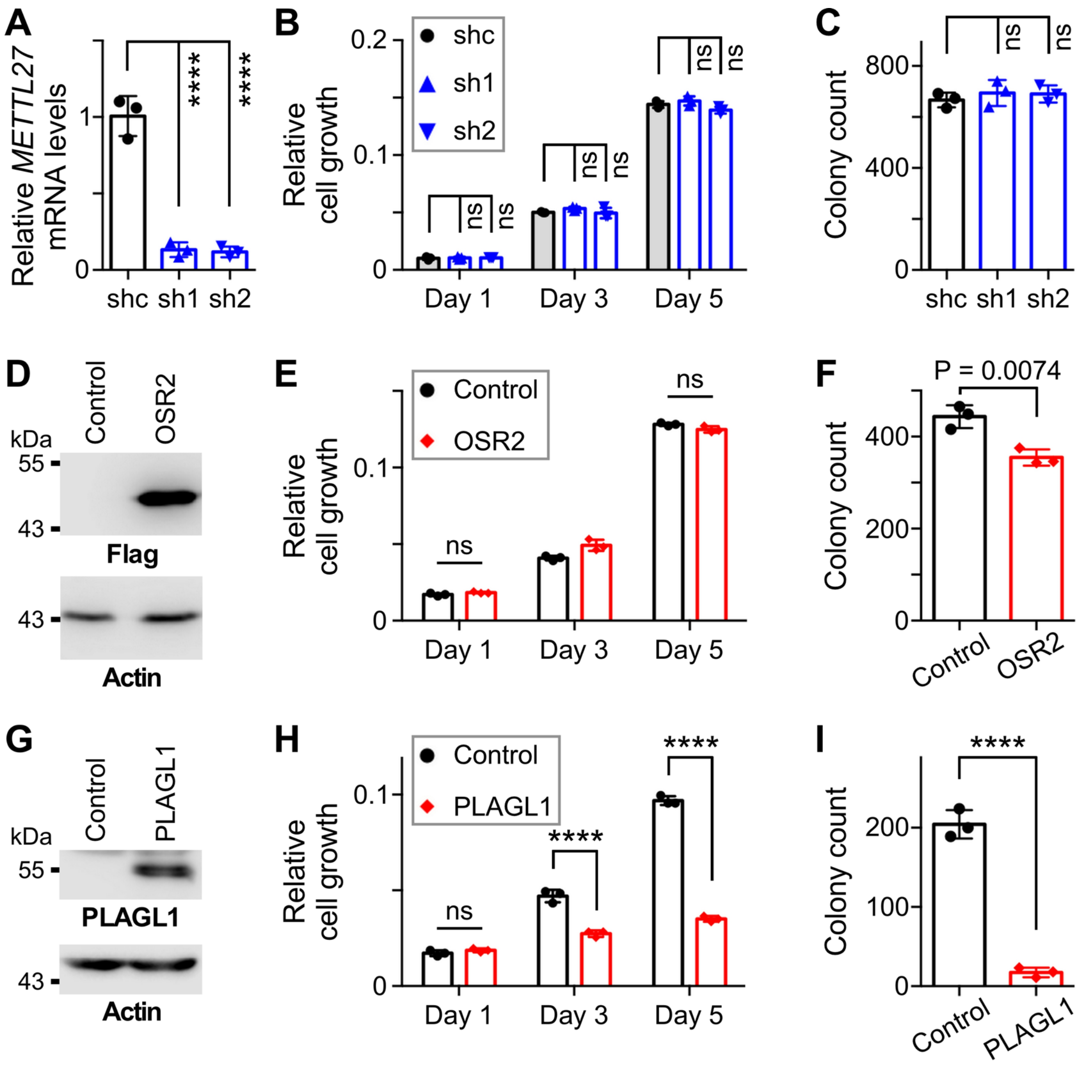

Supplementary Figure 6. Impact of METTL27, OSR2 or PLAGL1 on human DU145 prostate cancer cells. (**A**) Quantitative reverse transcription-PCR showing downregulation of *METTL27* mRNA upon expression of two respective shRNAs (sh1, sh2) compared to control shRNA (shc). The shRNAs were embedded into the *miR30* gene and targeted the sequences 5'-ATTGTCTACCTGTACCGAAAG-3' (sh1) and 5'-AGAAATGCCCTGCCTATTAAA-3' (sh2); one-way ANOVA (Tukey's multiple comparison test, n=3). (**B**) Corresponding cell growth (two-way ANOVA, Tukey's multiple comparison test, n=3) and (**C**) clonogenic activity (one-way ANOVA, Tukey's multiple comparison test, n=3). (**D**) Western blots of extracts from cells infected with control or Flag-OSR2 retrovirus. (**E**) Corresponding cell growth assay; two-way ANOVA (Sidak's multiple comparison test, n=3). (**F**) Respective clonogenic activity; unpaired, two-tailed t test (n=3). (**G**) Western blots of extracts from cells infected with control or Flag-PLAGL1 retrovirus. (**H**) Corresponding cell growth assay; two-way ANOVA (Sidak's multiple comparison test, n=3). (**I**) Respective clonogenic activity; unpaired, two-tailed t test (n=3). ****, P<0.0001; ns, not significant.

Supplementary Methods

Genotyping of CRISPR-modified DU145 cells

Genomic DNA isolated from DU145 prostate cancer cells was utilized in a PCR reaction with GoTaq DNA polymerase (Promega). The PCR program was as follows: 95°C for 2 min; 4 cycles of 95°C for 25 s, 66°C for 20 s (-1°C per cycle), 72°C for 25 s; 23-31 cycles of 95°C for 25 s, 62°C for 20 s, 72°C for 25 s; 72°C for 4 min. Primers used were (underlined bases are mutations introduced into the R427 clone):

K427-for: 5′-CTGCTGCTGTCCACAGCTCTAA-3′
R427-for: 5′-CTGCTGCTGTCCA<u>T</u>AGCTCTA<u>G</u>-3′
JMJD2D-2350-rev: 5′-CAGAAGCCTTGACAGGATGCTG-3′

Resultant 288 bp long PCR products were separated on 1.6% agarose gels and stained with ethidium bromide.

In addition, a 565 bp genomic DNA fragment was isolated after PCR with primers JMJD2D-2350-rev and JMJD2D-1786-for (5'-CTGCAACCTGAACGCTATGACCTG-3') with the same temperature cycling program as described above. After agarose gel electrophoresis, these fragments were cut out of the gel, purified with the QIAEX II Gel Extraction Kit (Qiagen) and then subjected to Sanger sequencing utilizing JMJD2D-1786-for as the sequencing primer.

Nucleotide sequences of PCR primers utilized for gene expression analysis

Gene	Direction	Sequence (5'→3')
CBLC	Forward	TCAGGCTACTGCTGAGGACT
	Reverse	CACATCTGGGTGCCCTGG
METTL27	Forward	TCCTTCGAGACCGTTTCGCT
	Reverse	GTGGCCACATCCTGGTCGT
COL4A5	Forward	TTCTCCTGAGAGACCGGCTT
	Reverse	ACCCATAGCAAGCCGCAG
GLIS3	Forward	CAAATCCTGGGAAAGGGGCT
	Reverse	TTGAGGCCGACTGACTTTCC
NPR1	Forward	TGTTTGAACTGAAGCATATGCGGG
	Reverse	GGCCGTCCACAGCTTTTTGG
OSR2	Forward	CCCCGGGTCTTTACACAGTC
	Reverse	GTAGAGAAAGCCCTCCCGC
PLAGL1	Forward	TCCCTGTCACTCAGTAGCCAA
	Reverse	CATCGTGGGCCTGGTTCTAC
RSPO3	Forward	TGGGTTACTATGCACTTGCGA
	Reverse	TCACAGTCAGCTTTGCATTTTGT
GAPDH	Forward	GAGCCACATCGCTCAGACACC
	Reverse	TGACAAGCTTCCCGTTCTCAGC
